# Craniofacial Changes Related to Maxillary Protraction Therapy in Mandibular Prognathism Associated With a Thyroglossal Duct Cyst: A Case Report

**DOI:** 10.1155/crid/1234184

**Published:** 2025-10-14

**Authors:** Shuzo Sakata, Ryo Kunimatsu, Ayaka Nakatani, Kotaro Tanimoto

**Affiliations:** Department of Orthodontics and Craniofacial Developmental Biology, Graduate School of Biomedical and Health Sciences, Hiroshima University, Hiroshima, Japan

**Keywords:** Angle Class III, cephalometry, child, malocclusion, orthodontics, retrognathia/therapy

## Abstract

Mandibular prognathism results from a complex interplay of genetic predisposition and environmental influences. In this study, we aimed to report the successful orthodontic management of a pediatric patient with mandibular prognathism that was potentially associated with a thyroglossal duct cyst in the floor of the mouth. This condition was treated with maxillary protraction therapy to improve the craniofacial morphology. A 9-year-5-month-old male patient presented with a major complaint of an anterior crossbite. Medical history revealed a congenital thyroglossal duct cyst in the floor of the mouth, which was surgically excised 8 months before the initial orthodontic consultation. Clinical and radiographic examination revealed severe mandibular prognathism characterized by maxillary hypoplasia and relative mandibular prognathia. We initiated maxillary protraction therapy using a protractor with a chin cup. Furthermore, significant skeletal improvements were observed following 30 months of active treatment: The SNA angle increased by 4.3°, the SNB angle decreased by 2.8°, the ANB angle increased by 7.1°, and the Wits appraisal improved by 14.1 mm. Long-term follow-up showed stable maintenance of these skeletal corrections at 4 years posttreatment. This case report suggested that a thyroglossal duct cyst in the floor of the mouth may be an environmental factor in mandibular prognathism pathogenesis. In addition, it demonstrates the clinical efficacy of maxillary protraction therapy in the orthopedic correction of Class III malocclusion during the active growth period.

## 1. Introduction

Mandibular prognathism is one of the most prevalent craniofacial skeletal discrepancies, with a multifactorial etiology involving genetic predisposition and environmental influences [1]. Extensive research has shown that genetic factors, including familial clustering patterns and ethnic predisposition, are crucial in the pathogenesis of mandibular prognathism [2–4]. Environmental factors implicated in its development include endocrine disorders [2], aberrant tongue posture [3], and macroglossia [4]. The treatment modalities for mandibular prognathism are age-dependent, and they encompass orthopedic intervention with maxillary protraction therapy during the active growth period and surgical orthodontic treatment for patients who are skeletally mature [5].

Thyroglossal duct cysts develop from persistent embryonic remnants of the thyroglossal duct during embryonic development. Furthermore, it generally manifests along the midline trajectory from the tongue base to the thyroid gland, including the floor of the mouth, submental region, and hyoid bone vicinity [6]. The majority of thyroglossal duct cysts occur in the infrahyoid region; however, intraoral presentation within the floor of the mouth represents a rare clinical entity [7]. Furthermore, mandibular prognathism secondary to dermoid cysts has been documented in previous case reports [8]; however, no literature has established an association between thyroglossal duct cysts and craniofacial skeletal development. In addition, only minimal investigation has occurred on the potential craniofacial morphological adaptations following surgical excision of such cysts.

In this report, we aimed to describe the craniofacial morphological outcomes following maxillary protraction therapy in a pediatric patient with mandibular prognathism that is potentially associated with a thyroglossal duct cyst in the floor of the mouth.

## 2. Case Presentation

**Figure 1:**
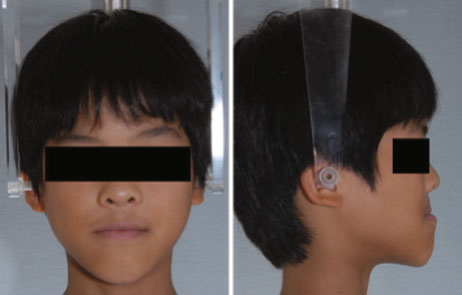
Pretreatment facial photographs.

**Figure 2:**
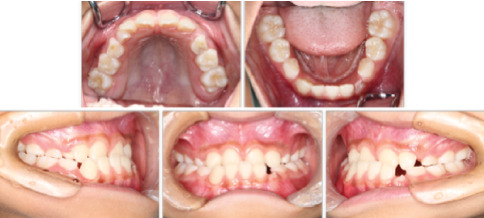
Pretreatment intraoral photographs.

**Figure 3:**
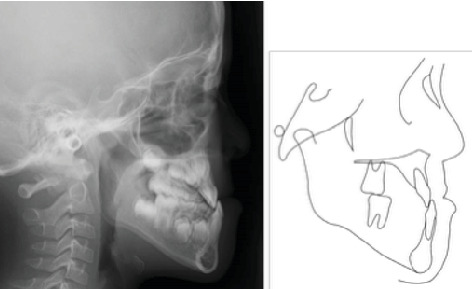
Pretreatment lateral cephalography and cephalometric tracing.

A 9-year-5-month-old male patient presented to the Orthodontics Department at Hiroshima University Hospital with a major complaint of anterior crossbite. Clinical examination revealed a concave facial profile with no apparent facial asymmetry (Figure 1). Medical and family histories were noncontributory for mandibular prognathism or craniofacial anomalies. Intraoral examination demonstrated an overjet and overbite of −2.0 and 4.0 mm, respectively, and an anterior crossbite (Figure 2). Dental development corresponded to Hellman's dental Age IIIb, with bilateral Angle Class III molar relationships. Functional examination showed anterior mandibular posturing during occlusion. Arch width measurements were within normal limits for both arches, with no observable posterior crossbite. Notably, there was a congenital absence of one mandibular incisor. Tooth size analysis revealed enlarged mesiodistal crown dimensions in both arches compared to normative values, suggesting potential maxillary crowding upon permanent dentition eruption. Lateral cephalometric analysis revealed severe mandibular prognathism, characterized by maxillary retrusion and mandibular protrusion, with an SNA, SNB, and ANB angle of 76.1°, 80.0°, and −3.9°, respectively, and a Wits appraisal of −11.9 mm (Figure 3). The gonial angle and Frankfort mandibular plane angle (FMA) were 130.8° and 29.7°, respectively, both indicating mild vertical skeletal excess. Dental compensation was evident, with lingual inclination of the maxillary and mandibular incisors resulting in an increased interincisal angle. Patient consent was obtained.

## 3. Medical History

**Figure 4:**
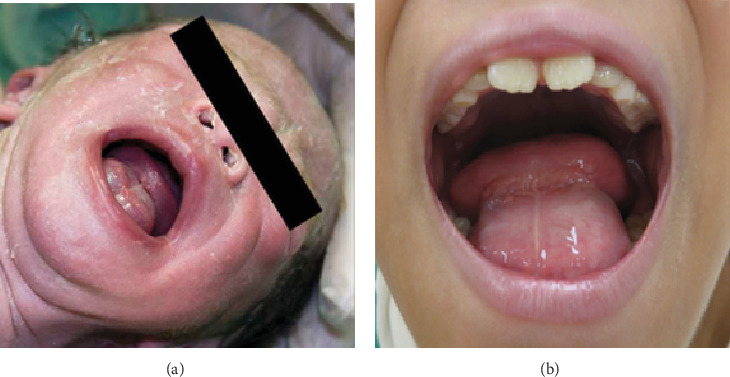
Photographs demonstrating a large mass in the floor of the mouth. (a) Photograph taken at birth. (b) Photograph taken before the cyst removal.

**Figure 5:**
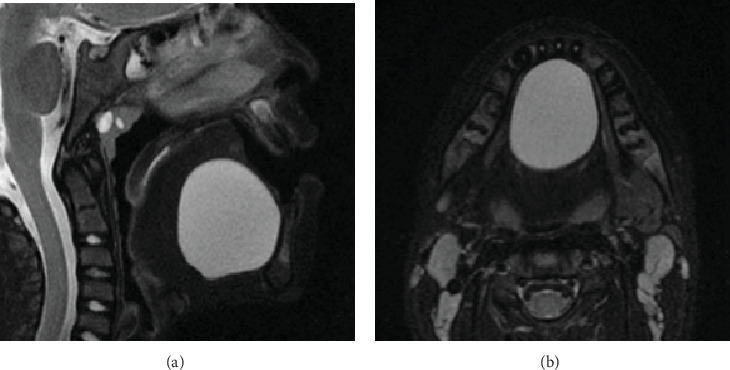
T2-weighted magnetic resonance imaging before the cyst removal. (a) Sagittal view. (b) Axial view.

**Figure 6:**
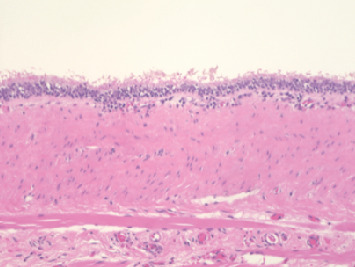
Hematoxylin and eosin–stained section of the excised cyst's lining.

The patient had a congenital thyroglossal duct cyst located in the floor of the mouth from birth (Figure 4a). The lesion remained asymptomatic and was managed conservatively as no functional concerns were reported by the patient or parents. However, the patient developed speech articulation difficulties attributed to the cyst 8 months before the initial orthodontic consultation, which required consultation with an oral surgeon (Figure 4b). T2-weighted magnetic resonance imaging demonstrated a large midline cystic lesion in the floor of the mouth with posterior tongue displacement (Figure 5). Surgical excision revealed serous fluid contents. Histopathological examination showed a cystic wall that was lined with nonkeratinized stratified squamous epithelium, with absent cutaneous adnexal structures (Figure 6), thereby confirming the thyroglossal duct cyst diagnosis. Complete healing without evidence of recurrence was observed at the 6-month postoperative follow-up.

## 4. Treatment Plan

Two treatment modalities were presented to the patient and parents following a comprehensive evaluation and discussion of the esthetic concerns regarding mandibular prognathism. The first option involved observation of growth until skeletal maturity, followed by surgical orthodontic treatment. The second proposed option was early orthopedic intervention using a protractor with a chin cup to modify craniofacial growth during the active growth period. Afterwards, the family opted for the second treatment approach following a thorough consultation. Informed consent was obtained with a clear explanation that maxillary protraction therapy may improve skeletal discrepancies; however, it would not preclude the potential need for future surgical intervention nor address anticipated maxillary dental crowding. Thereafter, orthopedic therapy was commenced with a complete understanding of the treatment limitations and expected outcomes.

## 5. Treatment Progress

**Figure 7:**
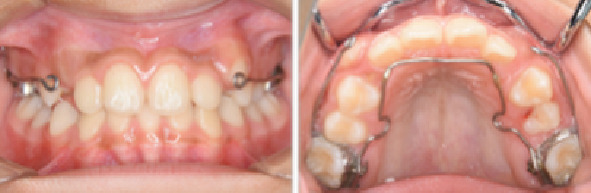
Intraoral photographs during protractor therapy.

**Figure 8:**
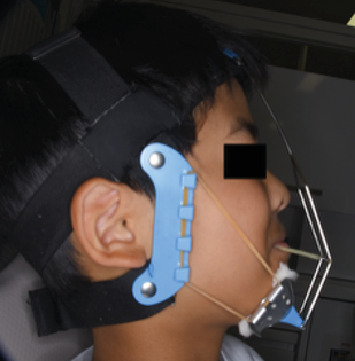
An extraoral photograph during protractor therapy.

**Figure 9:**
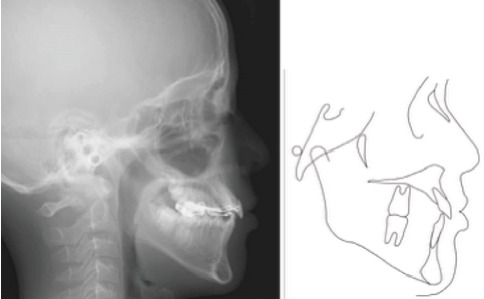
Posttreatment lateral cephalography and cephalometric tracing.

**Figure 10:**
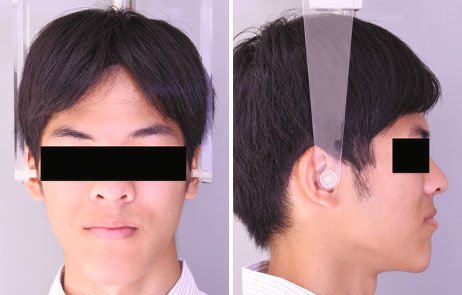
Four years posttreatment facial photographs.

**Figure 11:**
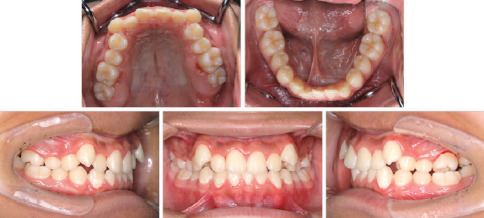
Four years posttreatment intraoral photographs.

**Figure 12:**
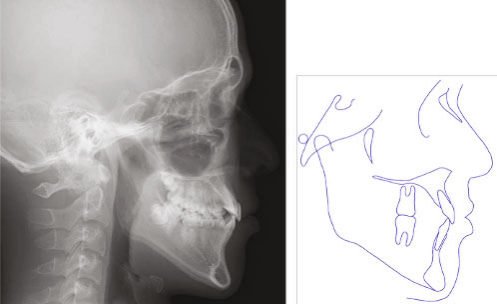
Four years posttreatment lateral cephalography and cephalometric tracing.

The maxillary first molars were banded, and a lingual arch was placed for anchorage reinforcement (Figure 7). Protraction hooks were welded to the molar bands' buccal aspect, positioned at the estimated center of resistance of the maxillary complex. Maxillary protraction was initiated 1 month postcementation, which involved applying 200–300 g of force per side via elastics connected to the protraction hooks. The force vector was directed 20° downward and forward relative to the occlusal plane (Figure 8). The patient was instructed to wear the protractor appliance for ≥ 12 h daily. Significant improvement in anteroposterior skeletal relationships was achieved following 30 months of active orthopedic treatment (Figure 9). The patient's compliance remained excellent throughout the treatment period. Posttreatment monitoring was conducted for 4 years to assess skeletal stability and potential relapse. The patient had an improved facial profile with corrected anterior relationship (Figures 10 and 11), as well as maintenance of favorable skeletal parameters at the 4th-year follow-up examination (Figure 12).

## 6. Treatment Results

**Figure 13:**
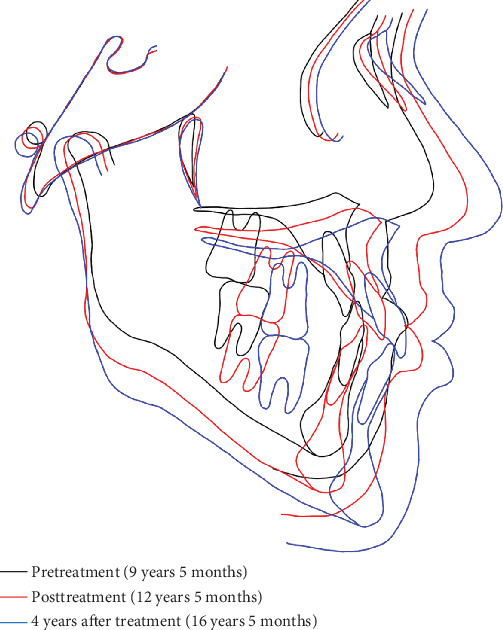
Superimposition of cephalometric tracing. Black line, pretreatment; red line, posttreatment; blue line, 4 years posttreatment.

Superimposition analysis of pre- and posttreatment, as well as 4-year posttreatment lateral cephalograms, demonstrated anterior–inferior displacement of Point A and posterior–inferior repositioning of Point B (Figure 13). The landmarks showed continued anterior and inferior movement compared with the baseline at the 4-year follow-up. The FMA and gonial angle remained relatively stable, with a slight decrease in the gonial angle measurement. Dental compensation was evident, with labial inclination of maxillary and mandibular incisors, and normalization of the interincisal angle. Significant skeletal improvements were achieved by maxillary protraction therapy: The SNA angle increased from 76.1° to 80.4°, the SNB angle decreased from 80.0° to 77.2°, and the ANB angle improved from −3.9° to 3.2°. Minimal skeletal regression was observed at the 4th-year follow-up; however, no clinically significant relapse of the mandibular prognathism occurred, and treatment outcomes remained stable (Table 1).

Assessment of upper airway dimensions and hyoid bone position at the initial examination.

**Figure 14:**
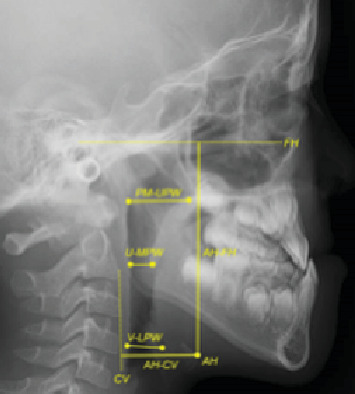
Reference points for upper airway dimensions and hyoid bone position measurements. PM, pterygomaxillary—projection of the tip of the pterygomaxillary fissure on the hard palate; UPW, upper pharyngeal wall—intersection of the palatal plane and posterior pharyngeal wall; U, tip of the uvula; MPW, middle pharyngeal wall—intersection of the line passing through U, perpendicular to the posterior pharyngeal wall; V, vallecula—the intersection of the epiglottis and the base of the tongue; LPW, lower pharyngeal wall—intersection of the line passing through V and perpendicular to the posterior pharyngeal wall; AH-FH, anterior hyoid–Frankfort horizontal plane—vertical position of the hyoid bone with respect to the Frankfort plane; AH-CV, anterior hyoid–cervical plane—horizontal position of the hyoid bone with respect to the vertebrae.

Space-occupying lesions in the floor of the mouth can cause displacement of adjacent soft tissue structures due to their mass effect [6]. This mechanical displacement can result in upper airway constriction and altered tongue posture and hyoid bone position [9, 10]. These anatomical alterations may have influenced normal craniofacial growth patterns adversely, potentially contributing to the pathogenesis of the skeletal Class III malocclusion observed in this case [11]. Therefore, lateral cephalometric analysis was performed to evaluate potential airway compromise and hyoid bone displacement in this case (Figure 14). Airway measurements revealed a nasopharyngeal, oropharyngeal, and hypopharyngeal dimension of 18.6, 8.8, and 13.0 mm, respectively. Hyoid bone position analysis showed an AH-FH (hyoid to Frankfort horizontal plane) distance of 73.9 mm and an AH-CV (hyoid to cervical vertebrae) distance of 27.6 mm. These upper airway dimensions and hyoid bone position measurements were within normal limits when compared to normative values [12].

## 7. Discussion

In this case, the congenital thyroglossal duct cyst in the floor of the mouth was hypothesized to be a contributing factor in mandibular prognathism pathogenesis, particularly considering the absence of familial history or genetic predisposition. A literature search revealed no previously documented association between thyroglossal duct cysts and mandibular prognathism. Large space-occupying lesions in the sublingual space may exert continuous anterior mechanical forces on the mandible during critical developmental periods, potentially influencing mandibular growth patterns [13, 14]. The baseline craniofacial morphology cannot be definitively established due to the cyst being congenital; however, it is plausible that this cyst may have contributed to mandibular prognathism development. Future prospective studies with larger patient cohorts presenting with cysts in the floor of the mouth should establish their role as environmental factors in craniofacial development. Cysts in the floor of the mouth may cause functional impairments, including dysphonia and dysphagia; nevertheless, asymptomatic lesions are often managed conservatively [15]. This case highlights the potential importance of early surgical intervention for preventing possible adverse effects on craniofacial growth and development.

Maxillary protraction therapy is an established orthopedic approach for managing skeletal Class III malocclusion in growing patients. The anteriorly directed force generated by elastic traction is transmitted through the maxillary first molars, promoting sutural remodeling and anterior displacement of the maxillary complex, thereby improving the anteroposterior skeletal relationship [16]. Secondary effects may include posterior repositioning and clockwise mandibular rotation. Similar skeletal adaptations were observed in this patient, indicating effective maxillary protraction therapy despite the potential environmental influence of the cyst in the floor of the mouth during craniofacial development. Notably, significant clockwise rotation of the mandibular plane angle was not observed, which may have been resolved by the concurrent decrease in gonial angle, suggesting favorable vertical growth control.

Extensive literature has documented mandibular incisor lingual inclination as a compensatory response to maxillary protraction therapy [17–19]. This case demonstrated labial movement of the mandibular anterior teeth. Furthermore, restoring normal tongue function following the cyst excision may have been instrumental in this atypical dental response. This observation indicates that the cyst in the floor of the mouth may disrupt the lingual and labial force equilibrium, thereby altering the natural inclination patterns of the anterior dentition.

The clinical course in this case demonstrated that maxillary protraction therapy can effectively improve skeletal Class III relationships despite potential environmental factors such as a large cyst, provided that surgical excision precedes orthopedic intervention. The absence of familial predisposition to mandibular prognathism in this patient may have contributed to the favorable skeletal response and long-term stability of the treatment outcomes.

In addition, airway morphology, respiratory patterns, and craniofacial growth and development are closely interrelated [11]. A large space-occupying lesion in the floor of the mouth may have contributed to this case's pathogenesis through altered respiratory function and tongue posture. Adenotonsillar hypertrophy, a primary cause of upper airway obstruction in the pediatric population, influences craniofacial development patterns [20]. Lateral cephalometric analysis has demonstrated adequate reliability in assessing upper airway dimensions and hyoid bone position in children and represents an appropriate methodology for evaluating this case [21]. Cephalometric analysis revealed no pathological airway constriction or anterior–inferior hyoid displacement in this present case [12]. Previous studies that examined upper airway changes following adenoidectomy demonstrated that postoperative dimensional improvements at 1 month remained stable through 1 year of follow-up [22]. In this case, 8 months had elapsed since surgical cyst excision, suggesting the stability of the upper airway normalization achieved through surgical intervention. These findings indicate that cyst-induced airway compromise can be effectively corrected through early surgical intervention, thereby emphasizing the necessity of timely treatment before irreversible skeletal adaptations occur.

This case report has several inherent limitations. First, the findings have limited generalizability as a single-patient case study, and conclusions drawn from one case cannot be extrapolated to the broader population. Future research should include larger cohorts, preferably comprising multiple cases of mandibular prognathism associated with thyroglossal duct cysts. This is to establish common patterns and treatment outcomes. Second, an absent control group limited our ability to determine whether the observed improvements were attributable solely to maxillary protraction therapy or to other contributing factors, including the natural resolution following cyst excision. In addition, potential confounding variables that may influence craniofacial and dental development were not evaluated comprehensively in this report. Future investigations should systematically assess and document a comprehensive range of potential confounders, including growth velocity, hormonal factors, and other environmental influences. This is aimed at providing an in-depth understanding of how various factors interact with skeletal malocclusion development and treatment efficacy.

## 8. Conclusions

In this case of severe skeletal Class III malocclusion, potentially associated with a congenital thyroglossal duct cyst in the floor of the mouth, we demonstrated that surgical cyst excision followed by maxillary protraction therapy during the active growth period can effectively improve anteroposterior skeletal relationships. This clinical experience suggests that cysts in the floor of the mouth may constitute an environmental factor contributing to mandibular prognathism development. In addition, early orthopedic intervention can achieve favorable outcomes with optimal patient compliance.

## Figures and Tables

**Table 1:** Changes in cephalometric measurements of pretreatment (T1), posttreatment (T2), and 4 years posttreatment (T3).

	**T1**	**T2**	**T3**
Facial angle (°)	89.8	89.2	91.4
Angle of convexity (°)	−7.5	2.9	−1.5
Gonial angle (°)	130.8	129.4	130
SNA angle (°)	76.1	80.4	82.1
SNB angle (°)	80.0	77.2	80.4
ANB angle (°)	−3.9	3.2	1.7
Wits appraisal (mm)	−11.9	2.2	−2.5
FMA (°)	29.7	29.1	27.4
U1 to FH (°)	104.8	114.4	113.5
FMIA (°)	83.6	68.9	70.1
IMPA (°)	66.7	82.0	82.5
Interincisal angle (°)	158.8	134.5	136.6

## Data Availability

The imaging data used to support the findings of this study are included in the article.
